# The Development of Frequency Tripler Based on Six-Anode Schottky Varactors

**DOI:** 10.3390/mi12121490

**Published:** 2021-11-30

**Authors:** Yuhang Li, Jin Meng, Dehai Zhang, Haotian Zhu

**Affiliations:** 1 CAS Key Laboratory of Microwave Remote Sensing, National Space Science Center, Chinese Academy of Sciences, Beijing 100190, China; liyuhang20212021@163.com (Y.L.); mengjin@mirslab.cn (J.M.); zhuhaotian@mirslab.cn (H.Z.); 2 School of Electronic, Electrical and Communication Engineering, University of Chinese Academy of Sciences, Beijing 100049, China

**Keywords:** Schottky varactor, frequency tripler, junction imbalance, cut microstrip stub

## Abstract

The development of a millimeter-wave unbalanced frequency tripler based on the nonlinear characteristics of planar Schottky varactors is presented. The entire module is designed by hybrid integration. A frequency multiplier circuit model was established to reflect the influence of diode parameters and the impedance matching on the multiplier in different frequency bands. The effect of junction imbalance on the output power of the frequency multiplier was investigated and the multiplier was improved based on the basic design. The addition of a cut microstrip stub in the improved diode unit reduced the impact of a power imbalance on frequency multiplier performance. The characteristics of the multiplier circuit were analyzed by the full-wave electromagnetic simulation of the three-dimensional structure and the harmonic balance simulation of the circuit. Test results showed that the peak output power of the improved frequency tripler was 12.6 mW at 277 GHz with an input power of 200 mW, an effective 12% improvement over the basic design.

## 1. Introduction

The development and utilization of the electromagnetic spectrum has always been a hot topic of concern in many research areas. As an important part of the electromagnetic spectrum, the terahertz frequency band in particular has received great attention in recent decades. Detectors used in astronomy, remote sensing, and atmospheric sciences in this frequency band are under continuous development. Its capabilities are increasing to meet the growing demand for powerful space-borne, ground-based telescopes, and facilities [1,2,3,4,5]. Many of these instruments have heterodyne receivers, which require a fixed, tuned local oscillator source. Therefore, the successful application of terahertz wave technology first needs to solve the problem of how to generate terahertz sources. The terahertz source based on the solid-state frequency multiplication circuit can work at room temperature and the structure is relatively compact, so it has become an important method of realizing the terahertz source at present [6]. Due to the increasing frequency of the current electronic system, if the terahertz source is generated in the form of a fundamental wave, the output power, stability, and other electrical characteristics required for operation may not be achieved at a very high frequency. At this point a low-frequency source with large output power and high stability is needed to obtain the required frequency through the way of nonlinear device frequency multiplication and meet the working requirements, which is the significance of the frequency multiplier. As the core device of the terahertz source, the performance of the frequency multiplier will directly affect the performance of the terahertz source. It has been improved and developed since the work of Penfield [7]. Several multiplier topologies have been developed based on the inherent isolation characteristics of odd and even harmonics; for instance, the balanced frequency doubler [8,9,10,11], frequency tripler [12,13,14,15,16], frequency quadrupler [17,18], and frequency quintupler [19,20] structures.

The balanced doubler in [8] achieved a typical testing efficiency of 7.5% at 190–225 GHz and maximum output power of 8.25 mW at 202 GHz with a pumping power of 85.5 mW. For triplers, a peak output power of almost 2.5 mW was achieved in [14] with 2.5% efficiency at 280–288 GHz. The quadrupler in [18] works at 325–351 GHz, with the output power being above 1mW between 334 GHz and 346 GHz, and the highest efficiency being above 3% with an input power of 100 mW. Finally, the quintupler in [20] delivers 2.8 mW of output power at 474 GHz with a 4% 3 dB bandwidth while using the silicon integrated heterostructure barrier varactor.

Since terahertz waves exhibit signal absorption peaks and atmospheric windows during atmospheric propagation, detection and communication studies can be carried out in specific frequency bands. The relatively transparent atmospheric window near 550 GHz can be used to detect and then analyze the characteristics of the atmosphere. Therefore, the 275 GHz frequency multiplier is designed to provide sufficient driving power for the 550 GHz sub-harmonic mixer at the back end, to ensure the normal operation of the whole system. In this paper, two 275 GHz frequency unbalanced triplers are designed by the hybrid integration method. The simulation of the 275 GHz tripler combines the three-dimensional electromagnetic simulation software and harmonic balance simulation tool so that the entire network can be analyzed and optimized. The overall circuit adopts the unbalanced structure, including a pair of symmetrically arranged planar Schottky varactors, and each diode chip is formed by three Schottky junctions connected in series in the same direction. This structure can effectively adjust the working state of the tube pair and provide an external bias voltage for the diode unit. To better reflect the effect of diode parameters and impedance matching on the frequency multiplier, a frequency tripler circuit model is established. In this way, we hope to provide a reference for the design of frequency triplers in different frequency bands. The effects of the indexes and parameters of the frequency multiplier are analyzed from the perspective of realizability. Symmetry condition is introduced in the design process, and the effect of junction imbalance on output power is analyzed according to the distance between the diode and air channel. According to the analysis results, the basic design was improved. The effect of a power imbalance on the frequency multiplier performance was reduced by adding a cut microstrip stub to the diode unit. Test results showed that the improved design effectively increased output power by 12%.

## 2. Design and Simulations

### 2.1. Frequency Tripler Circuit Model Analysis

**Figure 1:**
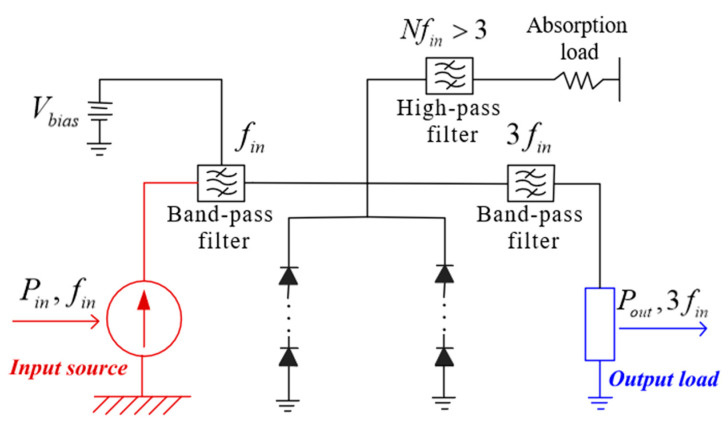
Frequency tripler circuit model.

In order to facilitate the design of frequency multipliers in different frequency bands in the future, a frequency multiplier circuit model is established as shown in Figure 1. The model involves trade-offs between major parameters. The parameters of Schottky diodes are based on existing processing technology and realizability. The filters in the circuit are all ideal filters and the required out-of-band rejection is achieved by setting the value of its S-parameter. Only the frequency tripler model is analyzed so that it can be easily compared. This model uses band-pass filters and DC feed components to perform the necessary signal separation. The circuit is connected to reverse bias to ensure that the diode in the circuit can work properly. Considering the design operating state of the frequency multiplier, the bias voltage unit is set to V, and the input power unit is set to mW. The high-pass filter and absorbing load in the model provide a return path for the harmonics greater than three.

Due to the different frequency bands, the value of the selected drive power is also different. According to the propagation theory of rectangular waveguides [21]:

(1) $$f=\frac{c}{\lambda_{c}}=\frac{c}{2a}$$

where $\lambda_{c}$ is the cutoff wavelength, a is the broad side of the standard waveguide, c is the propagation distance of the light wave in one second, and the cut-off wavelength of the rectangular waveguide is 2a. The cutoff frequency can be increased by reducing the width of the waveguide. Therefore, to ensure that only microstrip mode transmission exists in the circuit, the channel width of the circuit should be reasonably selected to cut off the waveguide mode of the corresponding working frequency when designing the frequency multiplier circuit. The channel width refers to the circuit channel width where the circuit substrate and diode are placed. The purpose is to ensure that only microstrip mode transmission exists in the circuit. Reducing the width of the circuit channel can cut off the high order mode at higher frequencies so that the mode in the circuit is simpler and the matching effect is better. However, the narrower channel width and diode length are not conducive to assembly and errors are common. Therefore, the number of anodes is limited by the physical size of the diode installation structure, which greatly affects the electromagnetic characteristics of the circuit in the terahertz range. The power processing capability deteriorates with the decrease in anode number, i.e., it will also affect the input power that the frequency multiplier can withstand and, to a certain extent, its frequency conversion efficiency. This model achieves the best frequency conversion efficiency and bandwidth by weighing the diode parameters and their best input and output embedded impedances under the available drive power and bias voltage conditions. It is worth mentioning that the input and output impedance values are obtained through simulation optimization when the best results can be obtained with the universal frequency multiplier model. Therefore, the most suitable tube with variable capacitance performance can be selected for circuit design, which provides a basis for the subsequent matching circuit design. The results of the frequency tripler circuit model are shown in Table 1.

Because the frequency multiplier designed in this paper is in the 275 GHz frequency band, the parameters of the Schottky varactor chip of ACST are as follows: zero bias capacitance $C_{j0}$ is 43 fF, series resistance $R_{s}$ is 4 Ω, ideality factor n is 1.1, and reverse saturation current $I_{sat}$ is 6 × 10^−16^ A. According to the empirical formula, the cut-off frequency of the planar diode can be obtained using [22]:

(2) $$R_{s}\cdot C_{j0}\ll \frac{1}{2\pi \cdot f_{c}}$$

where $f_{c}$ is the center frequency. According to the calculation, the cut-off frequency is 925 GHz, which meets the design requirements of the 275 GHz tripler in this paper. To realize the full-wave electromagnetic field analysis of the diode passive part, a lumped port with the thickness of the epitaxial layer is added under the anode column. This method is used to replace the nonlinear structure at the Schottky junction so the tube parameters can be introduced in the harmonic balance analysis.

### 2.2. Peripheral Circuit and Schottky Diode Unit Design

The peripheral circuit mainly includes a waveguide—microstrip transition, DC bias filter, low pass filter, and matching circuit. First, the design of the DC filter and low-pass filter corresponded to the ideal filter part in the frequency tripler circuit model established above. In the design process, the coupling effect of the equivalent capacitance and inductance of each order is used to improve the out-of-band suppression characteristics of the filter and the existing impedance discontinuity is taken into consideration. In order to prevent the falling edge of the frequency response from becoming too steep, the order of the equivalent lumped parameter filter should be controlled accordingly. The dimensions of the designed DC bias filter and low-pass filter are 1.98 mm and 0.914 mm, respectively.

Second, the design of the matching circuit needs to consider the characteristic impedance value, the equivalent dielectric constant, and the phase change. The purpose is to conjugate the input impedance and output impedance to the source and load impedance. This method makes the size of the matching circuit shorter than the initial design. The model of the frequency multiplier is designed with shorter and simpler matching branches and the results are close to the simulation results of the ideal frequency multiplier circuit. The degree of proximity of the two results is positively correlated with the fitting degree of the S-parameter. This part of the design is mainly realized by mutual iterative optimization of the three-dimensional electromagnetic simulation model and the circuit simulation model.

Third, the design of the waveguide—microstrip transition part is in the form of the E-surface probe. The fundamental wave signal is input through the standard WR10 waveguide in TE10 procedure and then converted into a quasi-TEM wave by the waveguide—microstrip E-surface probe. TE10 mode refers to the electromagnetic wave in the standard waveguide with a magnetic field component but no electric field component along the propagation direction. It is the most important mode in the rectangular waveguide as well as the primary mode. The rectangular waveguides operate in this mode in all practical applications. In the transmission line, the conductor band is the air above and the dielectric substrate below, so the real field in the microstrip line is a mixed TE-TM wave field. The longitudinal field component is caused by the edge field at the dielectric–air interface, which is smaller than the transverse field component between the conductor band and the grounding plate. Therefore, the transmission model property in the microstrip differs little from the TEM mode, which is called the quasi-TEM mode. Finally, the signal is output from the standard WR3.4 waveguide by an E-plane probe. In order to attenuate unnecessary harmonics, the input and output waveguide ports are widened and reduced. The dimensions of the broad side and narrow side of the input and output waveguide in this part are 2.54 mm × 0.52 mm and 0.65 mm × 0.3 mm, respectively. Furthermore, the corresponding suppression effect is achieved through this size, and at the same time, this processing can assist in completing the impedance matching.

Fourth, the Schottky diode unit is designed based on the diode layer structure. All pads of the selected diode chip are fixed on the microstrip circuit by a conductive adhesive. One end of the pad is connected to the microstrip circuit while the other end is connected to the cavity to form an RF/DC ground, which plays a role in heat dissipation. In order to match the length of the diodes, the semi-circular structure is left on both sides of the channel.

**Figure 2:**
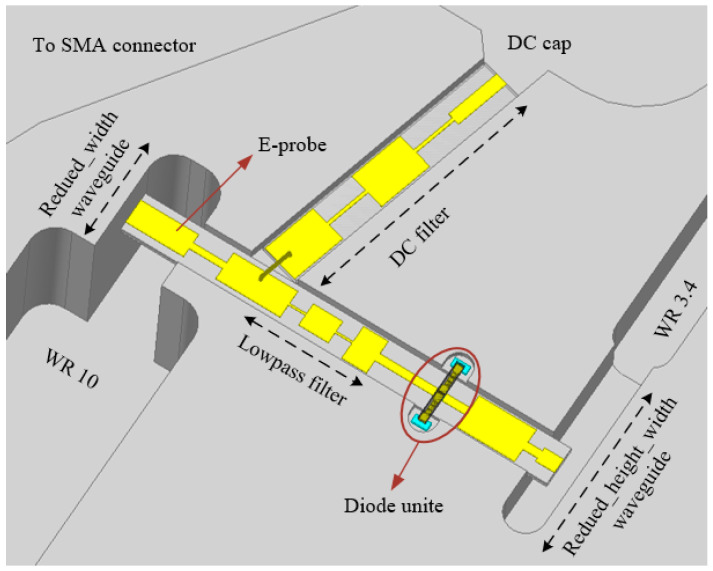
Overall three-dimensional electromagnetic frequency tripler model.

Finally, the overall three-dimensional electromagnetic model of the frequency tripler is constructed by combining the diode unit and the peripheral circuit, as shown in Figure 2. The final length of the circuit substrate obtained is 3.308 mm.

### 2.3. Anode Junction Imbalance Effect

**Figure 3:**
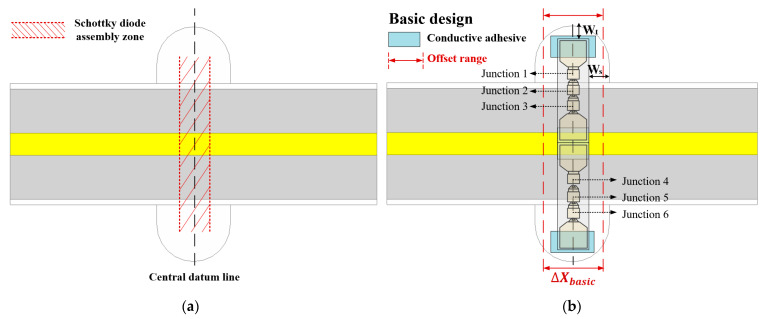

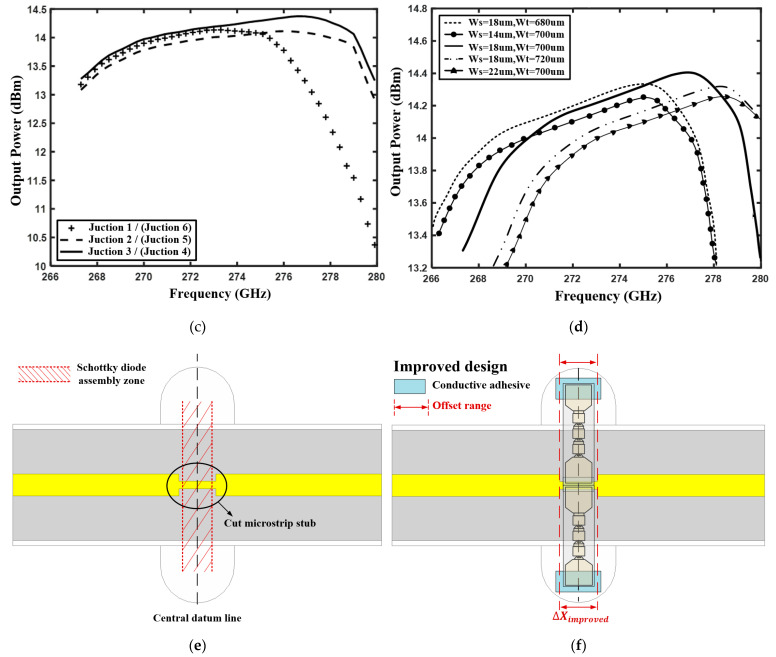
Junction imbalance simulation results, basic and improved design of Schottky diode unit. (**a**) Diode position in basic design; (**b**) Diode unit structure in basic design; (**c**) Output power imbalance of the 3rd harmonics at different junctions; (**d**) Output power vs. $W_{s}$/$W_{t}$ (at fixed junction); (**e**) Diode position in improved design; and (**f**) Diode unit structure in improved design.

The circuit model corresponding to Figure 2 is established by using a harmonic balance simulation tool and the output power is simulated and optimized in dBm. The assembly position of the diode is shown in Figure 3a. The center of the semicircular channel left on both sides is used as the reference line, and the two diode chips are placed upside down on the microstrip line using conductive adhesive. The sensitivity analysis of the multiplier is carried out in view of the errors produced by fabrication and assembly. The thickness of the conductive adhesive, the size of the input and output short-circuit ends, the offset of the substrate, and the distance between the diode and the air channel are analyzed. It was found that the distance between the diode and the air channel has the most significant impact on the performance of the frequency multiplier. In order to discover the mechanism of reduction, this part is analyzed emphatically.

The structure of the complete Schottky diode unit is shown in Figure 3b, which contains six junctions. As the six loads of the incident wave are in series, the input power will be divided into six parts and distributed to each junction. The output power of the 3rd harmonic is the combined power of six sources at the six junctions. At this point, it can be regarded as a power divider, which is defined as a device that can divide the energy of one input signal into multiple outputs of equal or unequal energy, and it can also combine multiple signal energies into one output. This part mainly realizes the function of signal power redistribution and recombination, but the balance degree of the output port directly affects the efficiency of the power synthesis. Therefore, the imbalance between the junction of the diode will have a great impact on the power of each junction at the fundamental frequency. At the same time, the matching network and diode position of the frequency multiplier will also affect the field distribution around the diode, which will reduce the power synthesis efficiency of the six-way synthesizer. Therefore, this paper considers the distance between the diode and the air channel and then analyzes the influence of the junction imbalance on the frequency conversion efficiency. The distance from the side of the semicircular channel is defined as $W_{s}$ and the distance between the diode and the tip of the semicircular channel is defined as $W_{t}$.

The 3rd harmonic output power of each junction is simulated in the 266–280 GHz range with a $W_{s}$ of 700 μm and a $W_{t}$ of 18 μm. The output power is divided into six parts, and each part represents a junction. When the input power is 23 dBm, the output power of each junction in a symmetric position has a good consistency, as shown in Figure 3c. That is to say, the symmetric condition method can be used to simplify the six nodes into three nodes. There is not only a significant difference in output power values among the three sets of junctions, but also the problem of frequency offset. Only the effect of the junction imbalance at junction 1/6 on the output power of the 3rd harmonic is shown to make a clear comparison. In consideration of the accuracy of fabrication and assembly errors, we let $W_{t}$ vary in the range of 685–715 μm and $W_{s}$ vary in the range of 14–22 μm. As shown in Figure 3d, the imbalance between the diode junctions is manifested in the output power changes with the changes of $W_{s}$ and $W_{t}$. This is due to the imbalanced incident power of each junction; the output power of the 3rd harmonic wave is imbalanced, which leads to the reduction of conversion efficiency and the frequency offset result. Furthermore, the results indicate that the change of $W_{s}$ value has a greater impact on the performance of the frequency tripler. In this case, the output power of each junction has a loss of nearly 0.4 dB.

### 2.4. Diode Unit Improved Design

The analysis of the effect of Schottky diode junction imbalance reveals that the combined efficiency of the six junctions is affected by the power distribution of each diode. The geometric parameters of the circuit also have a great influence on the junction imbalance, which directly affects the conversion efficiency. Therefore, the values of $W_{s}$ and $W_{t}$ should be carefully selected to reduce power imbalance, especially $W_{s}$. However, it can be seen from Figure 3b that the offset of the diode unit in the basic design has become more obvious during assembly. Meanwhile, the value of $W_{s}$ cannot be well controlled, where the offset range in the basic design is represented by $\Delta X_{basic}$.

Considering this situation, the Schottky diode unit is improved with a cut microstrip stub added at the connection between the conductive adhesive and the microstrip line, as shown in Figure 3e. Figure 3f is the improved design of the diode unit structure. Where the offset range in the improved design is represented by $\Delta X_{improved}$. Through the comparison between Figure 3b and Figure 3f, it can be found that $\Delta X_{improved}$ < $\Delta X_{basic}$, so the improved design can greatly reduce the offset of the diode during assembly. In other words, this stub fixes the position of the diode during assembly, so that the actual value of $W_{s}$ is closer to the design value, thereby reducing the effect of junction imbalance. The impedance discontinuity, characteristic impedance, and length of the cut microstrip subsection are added to the circuit model for optimization.

### 2.5. S-parameter Result Simulation

**Figure 4:**
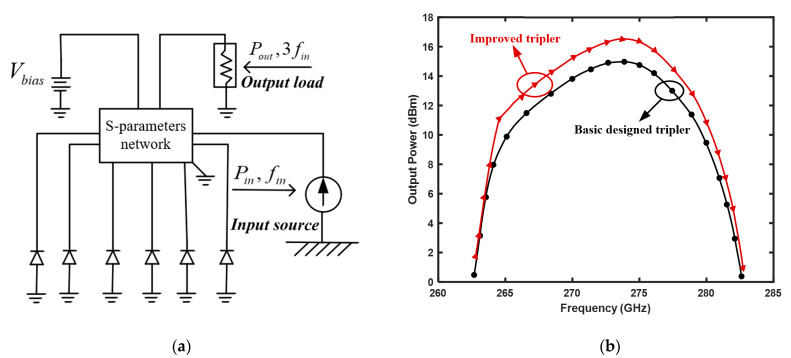
S-parameter network model and results. (**a**) Nine-port s-parameter network model and (**b**) Simulation results of basic designed frequency tripler and improved frequency tripler (Output power vs.$R_{s}$).

To verify the matching effect and frequency multiplication performance of the two frequency tripler, the Schottky nine-port S-parameter matrix of the above model was simulated in the nonlinear balance simulation software, as shown in Figure 4a. The w-band drive power at the front of the frequency tripler was fixed at 23 dBm. The simulation results of the basic design tripler and the improved tripler are shown in Figure 4b. It was reported that the output power of the improved tripler increased by 1dB compared to the basic design.

## 3. Measurements

### 3.1. Assembly and Test Setup

According to the above simulation results, two sets of frequency multipliers are fabrication and assembly. The entire outer cavity of the frequency multiplier is mirror-symmetrical, and a cavity with a cross-section of 1.4 mm and 1.4 mm is designed to place the chip capacitors. The DC filter circuit is connected with an SMA interface by bonding with an SMA connector (type KED204). Conductive adhesive is used to bond the quartz circuit to the corresponding channel of the lower cavity; the upper and lower cavities are fixed and closed with screws and pins. The installed frequency multipliers are 19.1 mm in length, 15.7 mm in width, and 24.8 mm in height, including input and output waveguide ports and DC bias ports.

The basic designed frequency tripler and improved frequency tripler were fabricated and assembled.

### 3.2. Results and Discussion

**Figure 5:**
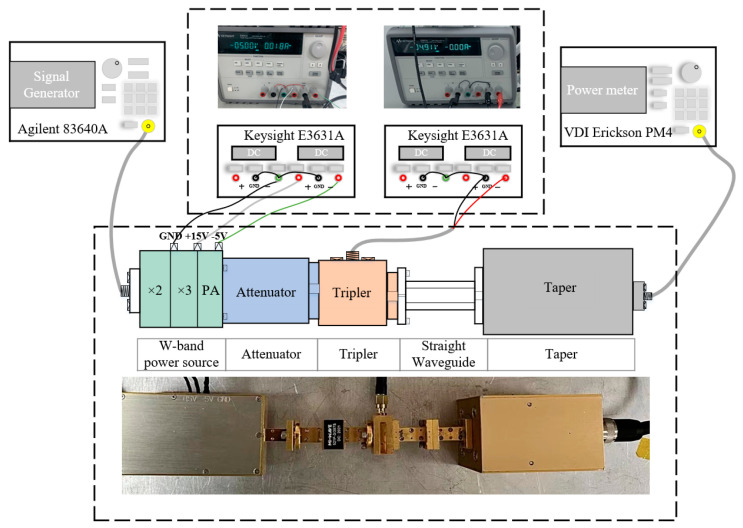
Block diagram of the test system.

The fabricated frequency tripler was tested and the test system is shown in Figure 5. It consisted of an Agilent analog signal generator (Agilent 83640A, Agilent Technologies, Santa Clara, CA, USA), two triple output DC power supplies (Keysight E3631A, Keysight Technologies, Santa Rosa, CA, USA), a W-band power source, an attenuator, the fabricated tripler, and a PM4 power meter (Charlottesville, VA, USA). The W-band power source included six frequency multiplication and amplification chips. One of the triple output DC power supplies provided the voltage of −5 V–+25 V for the W-band power source and the other triple output DC power supply provided the bias voltage for the fabricated frequency tripler. The signal generated by the power source was fed into the power amplifier after a six-fold frequency multiplication then connected to the developed frequency tripler and the final output power was measured by the power meter.

**Figure 6:**
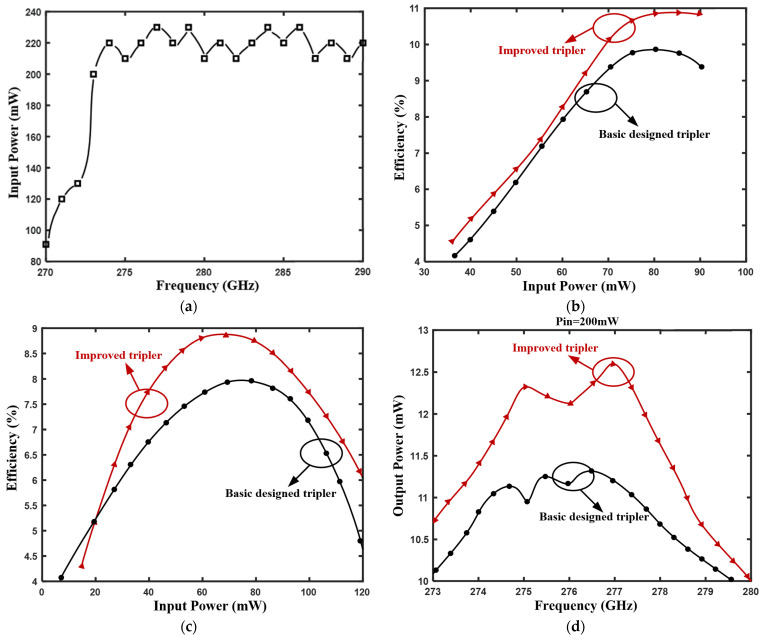
Test results (**a**) Fundamental drive source output power; (**b**) Efficiency vs. input power (at fixed frequency point 270 GHz); (**c**) Efficiency vs. input power (at fixed frequency point 271 GHz); and (**d**) Output power vs. frequency (fixed input power of 200 mW).

The maximum output power of the fundamental drive source used in the test was only 91 mW and 120 mW at 270 GHz and 271 GHz, respectively. However, an output power of more than 200 mW can be achieved after the 273 GHz frequency point, as shown in Figure 6a.

Because of the non-balanced circuit structure of the frequency multiplier, the efficiency is related to three variables: frequency, bias voltage, and input power. In the test, two of the variables were fixed to analyze the influence of other variables.

First, the frequency point and bias voltage value of the input signal were fixed and the input power changed. Figure 6b shows the change curve of conversion efficiency at 270 GHz with input power when the bias voltage was −5.6 V. The peak conversion efficiency of the improved tripler is 10.92% at this frequency point, and it is also the maximum conversion efficiency in the frequency range of 270–280 GHz. Figure 6c is the curve of the frequency conversion efficiency of the two frequency multipliers at 271 GHz with the input power when the bias voltage is −6.5 V. The frequency conversion efficiency increases with increasing input power until it reaches the maximum at a certain input power, at which point it tends to flatten because the diode is close to saturation at this time. When the input power exceeds a certain value, the efficiency decreases with the increase in input power. This test result is consistent with the theoretical analysis.

Second, the input power was controlled at a fixed value by adjusting the attenuator. When the input power was 200 mW, the output power varied with the frequency curve as shown in Figure 6d. The results show that when the fixed input power is 200 mW, the output power of the two frequency multipliers is greater than 10 mW in the frequency range of 273–280 GHz and the maximum value can reach 12.6 mW. The performance of the improved tripler was better than the basic design tripler. The test results confirm the previous analysis of $W_{s}$ and $W_{t}$; the differences in its value will affect the overall performance of the frequency multiplier. From the test results, it can be seen that the improvement of the diode unit reduces the effect of junction imbalance and increases the output power by 12%.

The comparison of the designed frequency tripler with the multipliers reported in the literature is shown in Table 2. Compared with the doubler [8], its power capacity is smaller than this work, owing to the smaller number of anodes. Compared with the triplers in [12,13,14,15], the proposed tripler had higher output power and efficiency. Although a high output power and broadband tripler work with monolithic HBV in [23], its efficiency is lower than in this work. However, integrated diode technology can be applied to high-frequency performance applications. From the above comparison, it can be concluded that the proposed frequency multiplier has a relatively excellent performance.

## 4. Conclusions

Based on the application of the terahertz frequency multiplier in the terahertz wave detection system, two unbalanced 275 GHz discrete variable capacitance triplers were designed, assembled, and tested in this paper. The aim was to provide the local oscillation source for the system, and together with the 550 GHz sub-harmonic mixer, to form the core components of the front end of the detection system. The basic design was improved by the addition of a cut microstrip stub in the diode unit. The test results show that the output power of the frequency tripler was increased by 12% with this approach. Both frequency triplers can meet the power requirements of local oscillators for the post-stage mixer within a certain bandwidth; as it plays an important role in the 550 GHz sub-millimeter receiver system. However, there are differences between the measured and simulation results. The test results have a drop of about 6 dB compared with the simulation result. There are two main reasons for this discrepancy: (1) Errors introduced in the process of machining and assembly; and (2) The package size of the diode used in the terahertz band is comparable to the signal wavelength. This leads to the inevitable high-frequency parasitic effects in the actual operation of the diode, which affects the high-frequency operating characteristics of the Schottky diode. In future research, the thermal coupling network and other high-frequency effects will be considered in the diode model. A thermal-coupled network and other high-frequency effects will be added into the diode model to reduce the gap between the simulation and measured results to improve the performance of the frequency multiplier.

## Figures and Tables

**Table 1:** Circuit model results.

Freq.3LO (GHz)	Z_diode_in_	Z_diode_out_	*C*_*j*0_(jF)	Anode Number	P_in_ (mW)	Bandwidth (GHz)	Efficiency (%)
100	52 + j.103	25 + j.66	102	6	400	35	58
200	22 + j.85	55 + j.88	65	6	200	32	42
300	95 + j.131	67 + j.113	38	6	200	30	33
400	80 + j.61	14 + j.90	15	4	100	32	25
500	120 + j.50	44 + j.52	10.5	4	100	31	20

**Table 2:** Comparison of reported frequency multipliers.

Ref.	Frequency (GHz)	Multiply Factor	Anode Number	P_in_ (mW)	P_out_ (mW)	Efficiency (%)
[8]	190–225	2	4	85.5	8.25	6–9.6
[12]	220–325	3	4	-	0.32–1.9	1.6–6.6
[14]	280–288	3	4	100	2.5	1.9–2.5
[15]	260–290	3	4	100	3.9–5.75	3.9–5.75
[23]	258–290	3	-	50–500	1–31	1.5–7
This work	270–280	3	6	120–200	9.9–12.6	5–10.92
